# Draft Genome Sequence of Entomopathogenic Bacterium *Photorhabdus temperata* Strain M1021, Isolated from Nematodes

**DOI:** 10.1128/genomeA.00747-13

**Published:** 2013-09-12

**Authors:** Gun-Seok Park, Abdur Rahim Khan, Sung-Jun Hong, Eun-Kyung Jang, Ihsan Ullah, Byung Kwon Jung, JungBae Choi, Na-Kyung Yoo, Keun-Joon Park, Jae-Ho Shin

**Affiliations:** School of Applied Biosciences, Kyungpook National University, Daegu, South Koreaa; Food and Biological Resources Examination Division, Korean Intellectual Property Office, Daejeon, South Koreab; Lifetechnologies Korea, LLC, NGS Field Application Specialist Team, Seoul, South Koreac

## Abstract

*Photorhabdus temperata* strain M1021 is an entomopathogenic bacterium belonging to the family *Enterobacteriaceae* and is symbiotically associated with nematodes. The draft genome sequence of *P. temperata* strain M1021 consists of 5,598,253 bp with a G+C content of 43.7%, and it has 6,120 protein-coding genes.

## GENOME ANNOUNCEMENT

The genus *Photorhabdus*, within the class *Gammaproteobacteria* and the family *Enterobacteriaceae*, currently includes species that are lethal pathogens of insects (1). *Photorhabdus* species live in a mutualistic association with entomopathogenic nematodes of the family *Heterorhabditidae* and are released from the gut of the nematode upon invasion into the insect hemocoel (2). The bacteria multiply and kill the host within 24 to 48 h because of the toxins produced by *Photorhabdus* bacteria, known as insecticidal toxin complex proteins (Tc), which exhibit oral as well as hemocoel toxicity (3). Three Tc components are required for full toxicity: TcdA-like, TcdB-like, and TccC-like components. Other toxins, including “makes caterpillars floppy” toxins (Mcf1 and Mcf2) promote the rapid destruction of the insect midgut, resulting in “floppy” caterpillars that suffer from a loss of body turgor (4). The *Photorhabdus* insect-related toxins (PirAB) are shown to be binary toxins with injectable and oral activity toward several species of insects (5).

*P. temperate* strain M1021 was identified and characterized by Jang et al. (6). The genome of this strain has been sequenced via the Ion Torrent Personal Genome Machine (PGM) sequencer system using a 316D sequencing chip (7). The sequence was assembled using MIRA 3.4.0. The assembled genome consists of 298 contigs (>418 bp), with a genome size of 5,598,253 bp at 31.50-fold coverage and a G+C content of 43.7%. The assembled contigs were submitted to the RAST annotation server (http://rast.nmpdr.org/) for subsystem classification and functional annotation. A total of 6,120 protein-coding sequences (CDS) were predicted, with 47% assigned to recognizable functional genes. There are 76 ribosomal genes, of which 71 are tRNAs and 1 and 4 are 16S and 23S rRNAs, respectively.

The complete gene cluster of insecticidal genes was predicted from the genome sequence. The genes for insecticidal proteins, such as TccA, TccB, TccC, and Mcf, have toxicity when injected into insects (E.-K. Jang, I. Ullah, S.-J. Hong, G.-S. Park, J.-H. Shin, unpublished data). Also, we studied phytohormone production and phosphate solubilization in *P. temperata* M1021 to produce the phytohormone indole-3-acetic acid (IAA) (8).

Knowledge of the *P. temperata* M1021 genome sequence will improve our understanding of insecticidal toxicity.

### Nucleotide sequence accession number.

The draft genome sequence of *P. temperata* strain M1021 has been included in the GenBank Whole-Genome Shotgun (WGS) database under the accession no. AUXQ00000000. The version described in this paper is the first version.
